# Recent Advances in Photodynamic Therapy for Deep-Seated Tumors with the Aid of Nanomedicine

**DOI:** 10.3390/biomedicines9010069

**Published:** 2021-01-12

**Authors:** Wei-Peng Li, Chia-Jui Yen, Bo-Sheng Wu, Tak-Wah Wong

**Affiliations:** 1 Department of Medicinal and Applied Chemistry, Kaohsiung Medical University, Kaohsiung 807, Taiwan; r091138@kmu.edu.tw; 2 Drug Development and Value Creation Research Center, Kaohsiung Medical University, Kaohsiung 807, Taiwan; 3 Division of Hematology and Oncology, Department of Internal Medicine, Graduate Institute of Clinical Medicine, National Cheng Kung University Hospital, Tainan 704, Taiwan; yencj@mail.ncku.edu.tw; 4 Department of Dermatology, National Cheng Kung University Hospital, College of Medicine, National Cheng Kung University, Tainan 704, Taiwan; 5 Department of Biochemistry and Molecular Biology, College of Medicine, National Cheng Kung University, Tainan 701, Taiwan; 6 Center of Applied Nanomedicine, National Cheng Kung University, Tainan 701, Taiwan

**Keywords:** photodynamic therapy (PDT), photosensitizer, hypoxia, metal–organic framework (MOF), pancreatic cancer

## Abstract

Photodynamic therapy (PDT) works through photoactivation of a specific photosensitizer (PS) in a tumor in the presence of oxygen. PDT is widely applied in oncology to treat various cancers as it has a minimally invasive procedure and high selectivity, does not interfere with other treatments, and can be repeated as needed. A large amount of reactive oxygen species (ROS) and singlet oxygen is generated in a cancer cell during PDT, which destroys the tumor effectively. However, the efficacy of PDT in treating a deep-seated tumor is limited due to three main reasons: Limited light penetration depth, low oxygen concentration in the hypoxic core, and poor PS accumulation inside a tumor. Thus, PDT treatments are only approved for superficial and thin tumors. With the advancement of nanotechnology, PDT to treat deep-seated or thick tumors is becoming a reachable goal. In this review, we provide an update on the strategies for improving PDT with nanomedicine using different sophisticated-design nanoparticles, including two-photon excitation, X-ray activation, targeting tumor cells with surface modification, alteration of tumor cell metabolism pathways, release of therapeutic gases, improvement of tumor hypoxia, and stimulation of host immunity. We focus on the difficult-to-treat pancreatic cancer as a model to demonstrate the influence of advanced nanomedicine in PDT. A bright future of PDT application in the treatment of deep-seated tumors is expected.

## 1. Introduction

The earliest light therapy for various skin diseases can be traced back to ancient Greece, Egypt, and India [1,2]. In 1907, Professor Hermann von Tappeiner coined the term photodynamic therapy (PDT) after incidental findings observed by his student Oscar Raab that fluorescence, a product of light and acridine together, was toxic to paramecium. Von Tappeiner and his partner, Dr. Jesionek, treated skin cancers, lupus of the skin, and condyloma with eosin dye with PDT, as reported in their book. This was probably the first human report of PDT. In 1841, a plant dye hematoporphyrin (Hp) showed more clinically satisfactory results and a higher level of biosafety than early photosensitizers (PSs) such as eosin, chinidine, and acridine [1]. In 1955, Samuel Schwartz successfully purified hematoporphyrin derivative (HpD), which shows a higher efficacy for tumors than Hp [1]. 

The modern era of PDT began in the 1970s in theUSA, after the first human report using HpD, later known as Photofrin, as a photosensitizer (PS) to treat skin cancers by Dr Thomas Dougherty working at Roswell Park Cancer Institute in Buffalo, NY [3]. Today, Photofrin is still the most commonly used PS globally. However, it has many disadvantages, including a long half-life lasting for weeks to months that leads to skin photosensitivity, and a small absorbance peak at 630 nm making it difficult to penetrate bulky tumors [3]. Since then, several hundred PSs have been developed to improve the efficacy of PDT to tackle cancer and many other diseases and infections.

The advantages of PDT include high selectivity to cancer cells and a high safety profile without long-term side effects. It can also be performed as an outpatient procedure, can be repeated if necessary, is less expensive than other treatments, and can be used as a curative or an adjuvant therapy. However, a successful PDT requires three essential factors, namely a well-designed PS, irradiation with an adequate wavelength covering the absorption peaks of the PS, and sufficient oxygen supply in the tumor microenvironment (TME) [4]. The currently available PSs have different unmet needs for clinical applications including most, if not all, absorption peaks being within the visible to near-infrared (NIR) wavelength of light, which limits the penetration depth to a few mm of the tissue. Therefore, PDT is only approved for the treatment of superficial and thin tumors [5]. Tumor hypoxia is a common phenomenon in the majority of malignant tumors due to rapid growth of cancer cells, which outgrows the blood supply and eventually leads to tumor resistance to PDT. 

Pancreatic cancer is the fourth highest cause of cancer-related death in the United States. The prognosis of pancreatic cancer is extremely poor, with almost all of the patients expected to die from the disease. One of the reasons is that the tumor is usually diagnosed at a late stage because it does not cause symptoms at the early stage. The only curable treatment is surgical resection. Unfortunately, only 15%–20% of patients are candidates for pancreatectomy at diagnosis. Even after a complete resection, the 5-year survival after margin-negative (R0) pancreaticoduodenectomy is approximately 30% for node-negative and 10% for node-positive disease. The global 5-year survival rate for pancreatic cancer patients is approximately 6% [6] despite the advancements in surgical technique, chemotherapy regimens, and the introduction of neo-adjuvant chemoradiotherapy.

Bown et al. initiated the first clinical trial of pancreatic cancer PDT in 2002. Sixteen patients with locally advanced pancreatic cancer were treated with percutaneous ultrasound and computed tomography (CT)-guided interstitial meso-tetrahydroxyphenylchlorin (mTHPC)-PDT with a prolonged median survival of 9.5 months (range 4 to 30, one patient alive at 31 months). Some novel photosensitizers have been developed and are under development to overcome the limitations of pancreatic cancer PDT [7]. 

Over the last two decades, the study and application of systems and manipulation of matter on the nanometric scale has become a mainstream academic field of interest [8]. Multifunctional nanoparticles (NPs) have been used as drug carriers for better targeting in cancer therapy via the enhanced permeability and retention (EPR) effect, enhancing imaging for diagnosis, additional functions in combination therapy, and their remotely controllable trigger of drug releases [9]. The concept and principle of nanotechnology has been recently utilized to overcome the limitation of PDT [10]. In this review, we use pancreatic cancer, a deep-seated tumor that is very challenging to treat, as a model to demonstrate how nanomedicine can be used as a potential tool to improve PDT.

## 2. The Principle of PDT and Limitations

### 2.1. Principle of PDT

**Figure 1:**
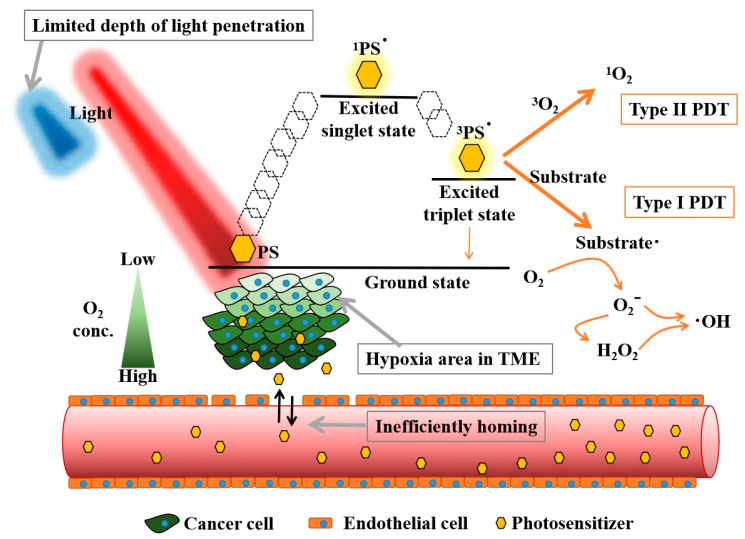
The principle of photodynamic reaction and its basic limitations applied in deep-seated tumor treatment. PS: Photosensitizer, TME: Tumor microenvironment.

Figure 1 depicts the mechanism of PDT. A ground state of PS absorbs photons and is transformed into its excited state (PS*), followed by the photophysical pathways of internal conversion and intersystem crossing to the lowest excited singlet state or lowest excited triplet state. These lowest excited states of PS* can directly revert to the ground state by radiation emission. The lowest excited triplet state PS* has a longer lifetime for energy transfer to biological substrates to form reactive oxygen species (ROS) (type I reaction) or electron transfer to oxygen to form the singlet oxygen (^1^O_2_) (type II reaction) (Figure 1) [11]. Type II reaction is the major mechanism in PDT in cancer therapy. Nevertheless, the proportion of type I/type II reaction is dependent on the PS used in PDT [5].

### 2.2. Limitations of PDT Application in Oncology

An ideal PS should contain some intrinsic features such as minimal toxicity to normal tissue at a therapeutic dose, a properly metabolizable rate from the body, defined accumulation in target cells, activation by a longer wavelength of light, which is usually above 550 nm; and high yield of reactive oxygen species (ROS) after irradiation [1]. A successful PDT treatment requires optimal light energy to destroy tumor cells with sufficient PS distribution and plenty of oxygen in the TME (Figure 1) [12,13,14]. 

In conventional PDT, visible and near-infrared light are usually used as excitation wavelengths depending on the absorption peaks of a PS. These wavelengths of light penetrate only few mm of the skin (or tumor), which is one of the major drawbacks of PDT in treating a bulky tumor or a tumor inside the body such as pancreatic cancer [12]. 

The presence of oxygen in the TME directly affects the ROS generation level in PDT. However, tumor hypoxia is common in solid tumors, especially in the core of the tumor [13]. The tumor core contains the cancer stem cells and resistant cancer cells with a lower level of oxygen and nutrients due to limited vascular supply. These cells have a poor response to radiation, chemotherapy, and especially to the PDT, which requires oxygen [13]. The low efficacy of PDT in solid tumor treatment causes the resurgence of malignant stem cells [15]. Dose–effect is a vital pharmacokinetic consideration when evaluating the dose-response related to efficacy and toxicity [16]. In designing an ideal PS, it is necessary to consider the intrinsic physicochemical properties, drug internalization to a cancer cell, PS accumulation in the tumor site, lowest effective dose, harmless metabolism pathway, reasonable half-life, and the potential cytotoxicity to normal cells [14].

## 3. Innovative Nanotechnologies to Improve PDT Treatments

**Figure 2:**
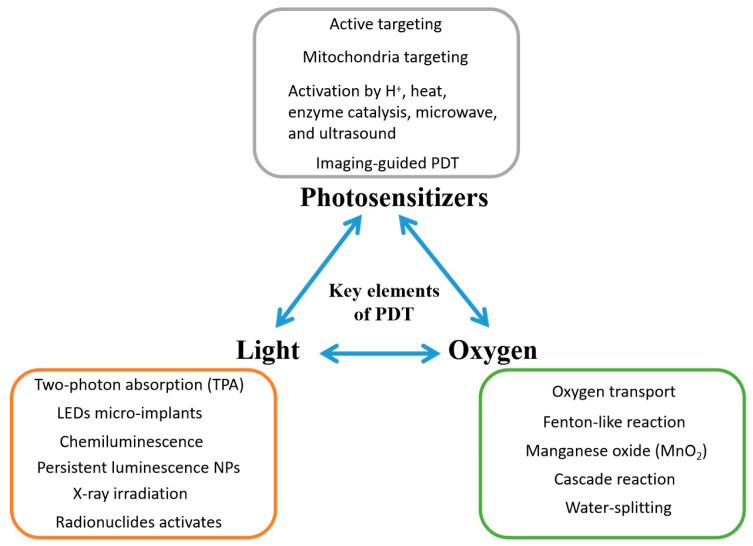
Strategies for improving photodynamic therapy with nanoparticles. NPs: Nanoparticles.

With the advancement of nanotechnology, the application of PDT in deep-seated tumors has become possible. New novel NPs can serve as better PS carriers, shift the PS to a higher absorption peak, and work better in a hypoxic TME. Figure 2 shows how NPs can help to improve modern PDT in different ways. Table 1 reveals the various nanomaterials with particular properties providing helpful functions to relieve these limitations of PDT in treating deep-seated tumors in clinical condition. 

### 3.1. Feasible Strategies to Initiate PSs in Deep-Seated Tumors

#### 3.1.1. Improve the Light Penetration and Activation via Two-Photon Absorption

The Achilles’ heel of all photoactivated therapies is the light penetration of the target tissue [12]. Biological windows I and II of NIR ranges from 750 to 950 and 1000 to 1350 nm, respectively, with a better tissue penetration and biosafety than the light at the UV and visible range below 650 nm [17,18]. NIR light emitted from an 830 nm LED can penetrate formalin fixed soft tissue, bone, and the brain [19]. Recently, the third NIR biological window signifying the range of wavelengths from 1550 to 1870 nm has been developed and can achieve a superb light penetration depth into the human body [20]. 

**Figure 3:**
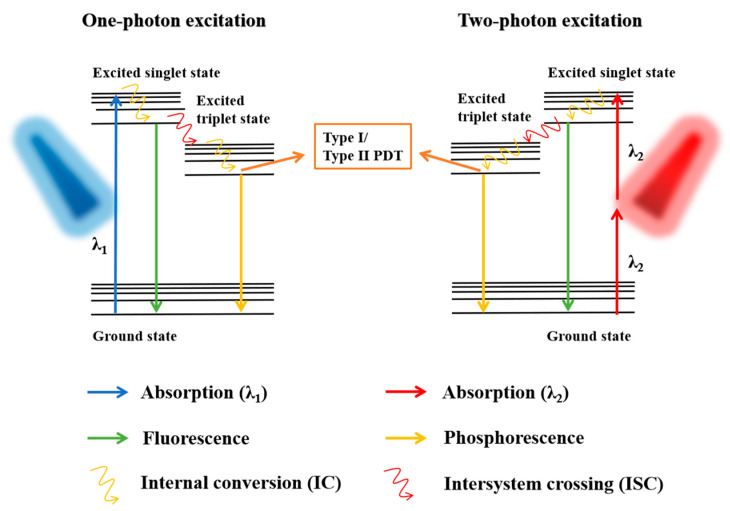
Photosensitizer activation through one-photon excitation and two-photon excitation (TPE). The TPE requires two photons of approximately half the energy compared to the energy needed in one-photon excitation.

Another feasible technique is two-photon excitation (TPE) which allows the use of a lower energy irradiation to activate PSs with two-photon absorption (TPA), resulting in a significant improvement in the light penetration depth throughout the body and reducing the additional photodamage to healthy tissue (Figure 3) [21]. A high TPA cross-section (σ_2_) is the most crucial requirement for two-photon-activated PDT [22]. Qiu et al. reported that fluorinated ruthenium (II) PSs activated by a low power NIR light were highly effective in TPA for PDT ablation of cell membranes and mitochondria in cancer cells [23]. When a common porphyrin PS is conjugated with an AceDAN donor, it accepts the energy to form a fluorescence resonance energy transfer (FRET) system, which exhibits remarkable two-photon-activated PDT performance [24]. Several PS-based self-assembly NPs showed larger TPA σ_2_, revealing the advantage of PSs nanoization in TPA and, together with an additional function of the EPR effect for tumor accumulation, an enhancement of PDT efficacy [25,26].

Aggregation-induced emission (AIE) refers to a class of luminescent materials, which are weakly or non-emissive in the form of isolated molecular species but emit strong fluorescence in the aggregated and solid states [27]. NP-modified PSs with the AIE effect showed potential for use in two-photon-activated PDT with the fluorescence imaging function [28,29,30]. Sun et al. used polyethylene glycol (PEG)-modified nanographene oxides to embed AIE NPs, presenting good stability in buffer solution and a typical feature of TPA [28]. Zhuang et al. designed and synthesized two new two-photon AIE luminogens with a stiff D−π−A skeleton, termed the TPPM and TTPM, showing augmented properties in AIE with a high fluorescence quantum yield up to 290-fold [29]. Outstanding TPA-PDT polymer dots with AIE were reported by Wang et al. [30]. The Au cluster AIE PS also has significant TPA properties and a strong type I reaction, thereby showing potential for two-photon-activated PDT application [22,31,32]. An ultrahigh TPA PS was observed from dihydrolipoic acid-coated gold nanoclusters [22]. Semiconductor material ZnTPyP@TiO_2_ nanocomposites were synthesized by Liu et al. with strong TPA ability under 800 nm irradiation [33].

#### 3.1.2. Direct Implantation of a Mini Light Source into a Tumor

Directly implanting a lamp or illuminator at the solid tumor site seems to be a promising approach to overcome light penetration depth from an external light source. Liu et al. implanted micro light-emitting diodes (LEDs) into tumors for PDT driven by body motion [34]. An implantable ultrasonically charged micro light source for PDT was reported by Kim et al. [35]. The molecular or nanoscale illuminators exhibit multiple benefits compared to micro-implants, including the non-invasive procedure, increased comfort during treatment, accumulation of illuminators in tumors with long-term retention, and the ability to equip illuminators with a PS or drug. Heptamethine aminocyanine dye and the nanocrystallized organometallic phosphorescent have demonstrated excellent performance with a long-lived excited triplet state, thus revealing the potential use of ultralong phosphorescence for afterglow imaging and activating PS to PS* [36,37]. 

#### 3.1.3. Self-Lighting PDT

Another strategy to overcome light penetration in PDT is through the creation of self-exciting nanoplatforms, which do not need an external light source for PS activation. These platforms are driven either from a specific chemical reaction or enzyme catalytic biochemical reaction in a tumor [38]. Wu et al. formulated a chemiluminescence resonance energy transfer (CRET) strategy for solid tumor PDT by using a nanoreactor in which the NP encapsulated bis[3,4,6-trichloro-2-(pentyloxycarbonyl) phenyl] oxalate (CPPO), poly[(9,9’-dioctyl-2,7-divinylene-fluorenylene)-alt-2-methoxy5-(2-ethyl-hexyloxy)-1,4-phenylene] (PFPV) and the tetraphenylporphyrin (TPP) as a PS. The self-lighting took place using CPPO oxidation which catalyzed endogenous H_2_O_2_ in the TME. The intermediate chemical was unstable, which transferred energy to excite PFPV (Cerenkov resonance energy transfer, CRET) [39]. Yang et al. utilized luciferase-exposed PS-loaded poly(lactic-coglycolic acid) (PLGA) nanoparticles to activate the bioluminescence resonance energy transfer (BRET)-mediated PDT for tumor treatment [40].

In recent years, persistent luminescence nanoparticles (PLNPs) composed of ZnGa_2_O_4_:Cr (ZGC) have shown promising results in PDT. This material is referred to as Night Pearl because the bulk luminous material is as bright as an illuminating pearl at night in an ancient Chinese legend. These NPs have a better EPR effect and emit NIR (~700 nm) in the tumor site over the course of a few minutes to a few hours [41,42]. PLNPs have been embedded in the biocompatible alginate-Ca^2+^ hydrogel [43] and PLGA oleosol [44] as injectable implants used for long-lasting light supply in tumors.

#### 3.1.4. Activation with X-ray

X-ray photons are considered a powerful light source, with superb penetration in solid tumor treatment [45]. It is well recognized that the heavy elements (high-Z elements) are sensitive to X-ray photons due to larger overall photoabsorption cross-sections and a significant photoelectric effect compared to low-Z elements, named the high-Z effect. After exposing high-Z NPs to X-ray, scattered X-rays, photoelectrons, and fluorescence photons were generated, which in turn triggered the surrounding PS to form abundant ROS [46]. Song et al. proposed that W(VI)-doped ZGC nanocrystals were activated by low-dose X-ray irradiation (0.8 Gy) to form persistent NIR light emission [47].

Diverse radiation-sensitive materials, including CaWO_4_ NPs, SrAl_2_O_4_:Eu^2+^ NPs, Cu NPs, Au nanorods, and Au_8_ nanocrystals, constructed by mid- or high-Z elements showed potential in X-ray-triggered persistent luminescence-mediated PDT [48,49,50,51,52]. Deng et al. proposed a platform using PLGA vesicles to carry PS verteporfin and gold NPs (2–5 nm) together for efficient energy transfer between the PS and Au NPs. The NPs showed excellent results in PDT with a low dose of radiation (4 Gy) [53]. PS-modified α-Zn_2_SiO_4_-doped silica NPs were also used to emit X-ray-excited optical luminescence (XEOL). The activation was achieved by very-low-dose radiation (1 Gy). The combination of radiotherapy and X-ray-induced PDT showed significant efficacy in inhibiting deep-seated tumors [54].

Sun et al. designed AIE gold clustoluminogens to achieve a low-dose X-ray-induced PDT with generation of abundant ROS and with almost no side effects [55]. Lanthanide-doped nanoparticles also revealed the potential for use in X-ray-induced PDT [56,57,58]. Synergistic effects in tumors were found for activatable NPs with radiation and reactive oxygen species-releasing nanostructures designed by Cheng et al. [59].

Radionuclide activation is able to emit Cherenkov radiation (CR), the intense blue light of which is applied as an endogenous light source in CR-induced PDT to the deep-seated tumor. Water-soluble sulfonato-aryloxy-Zn (II)-phthalocyanine NPs receive the CR by four aryloxys (radio antenna), leading to an energy transfer to initiate the Zn (II)-phthalocyanines (which serve as a PS) for further ^1^O_2_ production [60]. Ni et al. used magnetic nanoparticles (MNPs) to carry radionuclides to activate ^89^Zr and PS molecules, which showed a 5-fold accumulation of MNPs at the tumor site by magnetic targeting [61].

### 3.2. Modulation of Oxygen Concentration in Tumor Microenvironment

The oxygen level at the tumor microenvironment is a significant factor affecting the sufficient singlet oxygen production during PDT [13]. Many scientists are developing different feasible strategies to modulate the oxygen supply in cancer cells for tumor hypoxia relief, thus boosting the ^1^O_2_ generation in TEM during PDT.

#### 3.2.1. Applying the Metal–Organic Framework for Oxygen Delivery

The porous metal–organic framework (MOF) nanomaterial constructed by metal complexes and organic ligands under an orderly arrangement has emerged as a candidate in the field of nanomedicine, showing various applications such as gas loading, photo-catalysis, and drug delivery, as well as exhibiting good biocompatibility and biodegradability [62]. Various MOFs assembled by different metal clusters and bridging molecules offer unique characteristics, and the MOF is thus one of the perfect candidates in PDT application.

In 2018, Gao et al. applied a zirconium (IV)-based MOF to store oxygen after loading oxygen and anchoring the indocyanine green (ICG, an NIR organic dye) on the MOF. This as-prepared material was encapsulated inside the cell membrane to form a biomimetic nanoplatform releasing NIR light-responsive oxygen [63]. Ren et al. indicated that the zeolitic imidazolate framework-67 (ZIF-67)-based MOF has a high reactivity and can produce endogenous hydrogen peroxide (H_2_O_2_) inside cancer cells [64].

Some PSs have been used as the organic ligands in MOF preparation to form the photosensitizable MOF [65,66,67,68]. Integrating tetrakis(4-carboxyphenyl) porphyrin (TCPP) into a Zr-based MOF gives the ability to generate ^1^O_2_ with a low power laser at 650 nm (0.1 W/cm^2^) [66]. Wang et al. prepared ultrasmall porphyrinic MOF nanodots (4 nm), which not only showed a significant photoresponsive cell-killing effect but also led to an efficient elimination from the body through renal clearance [68].

#### 3.2.2. Fluorine-Contained Nanocarrier for Oxygen Delivery

Perfluorocarbon (PFC) consists of a carbon skeleton and high electronegative fluorine and exhibits an excellent oxygen affinity, representing a suitable material for oxygen transport [69,70,71]. A case using pH-sensitive PFC-modified nanoparticles to load oxygen and a PS, IR780, has been reported by Ma et al. [69]. An amphiphilic/fluorous random copolymer was used to construct micelles showing good O_2_ carrying ability, thereby revealing satisfactory photocytotoxicity [70]. Likewise, Hu et al. indicated that PFC and oxygen co-loaded hyaluronic acid (HA) vesicles grafted with chlorin e6 (Ce6) by reducible disulfide bonds revealed excellent performance in carrying oxygen. These vesicles could be destroyed by glutathione (GSH), which is enriched inside cancer cells to release oxygen in the hypoxia TEM [71].

#### 3.2.3. Decomposition of Endogenous Hydrogen Peroxide into Oxygen

Decomposing endogenous hydrogen peroxide in cancer cells through the enzyme-induced catalytic reaction is a reliable way to raise the oxygen level. Various nanomaterials, such as MOF, black phosphorus (BP), HA-based NPs, and fluorinated polyethyleneimine (F-PEI) NPs, were used to load catalase (CAT), by which H_2_O_2_ could be decomposed into O_2_, thus showing an outstanding PDT efficacy [72,73,74,75]. Some strategies using nanomaterials with CAT-like catalysis activity were developed and revealed excellent performance in the decomposition of hydrogen peroxide and oxygen generation in cancer cells [76,77,78]. A 2D MOF nanosheet assembled by transition metal ions (Sm^3+^), platinum, and TCPP presented catalase-like activity for sufficient oxygen supply during PDT [79]. V_2_O_5_ nanoparticles exhibited their potential to regulate the oxygen amount in the TME through their intrinsic peroxidase-like activity to decompose H_2_O_2_ to O_2_ [80].

Recently, manganese oxide (MnO_2_) has been observed to be a critical candidate for hypoxia relief in the TME through its reaction with endogenous H_2_O_2_, in which the MnO_2_ can be decomposed into Mn^2+^ and oxygen under an acidic microenvironment. Various nanoparticles, such as gold nanocages, BP-based nanocomposites, semiconducting polymer nanoparticles (SPNs), and poly(amidoamine) (PAMAM) dendrimer-based NPs, integrated with MnO_2_, were applied to sustain the oxygen inside cancer cells [81,82,83]. Magnetic Fe_3_O_4_ NPs coated with mesoporous MnO_2_ nanosheets were used to form magnetic manganese oxide sweetgum-ball nanospheres (MMOSs), which showed remarkable material accumulation at the hypoxia tumor by magnetic field attraction and provided oxygen evolution through the self-decomposition of the MnO_2_ shell at the TME [83].

Carbon dots (CDs) have been reported as a suitable candidate for PDT due to their excellent ability to efficiently produce singlet oxygen. Jia et al. used manganese (II) phthalocyanine as a precursor to prepare the Mn-CDs, revealing its capability to be an acidic H_2_O_2_-driven oxygenator to further boost the PDT effect on the solid hypoxic tumor [84]. A photothermal material using Au@m-SiO_2_ nanocube-loaded doxorubicin (DOX) as the chemotherapeutic drug grafted with Mn-CDs shows multiple applications for hyperthermia, chemotherapy, and oxygen-evolving in situ PDT under bi-irradiation at 635 and 808 nm [85].

#### 3.2.4. The Water-Splitting System for Anti-Hypoxia Effect

Water-splitting materials used in the biomedicine field have attracted widespread attention. Jiang et al. developed graphdiyne oxide (GDYO) nanosheets that are able to split water for sufficient oxygen generation in the TME upon irradiation at 660 nm [86]. Coating the mesoporous carbon nitride (C_3_N_4_) nanoparticle shells showed excellent performance in intracellular water splitting, thereby raising the O_2_ level for attenuation of the hypoxic state [87].

### 3.3. Enhancing Targeting on Cancer Cells

Abnormal angiogenesis around the solid tumor causes the large gaps (~100 nm) between endothelial cells in blood vessels, thus resulting in a higher permeability of nanoparticles into the tumor site. The quick growth of the tumor leads to loss of lymphatic reflux, thus making those trapped nanoparticles stay in the tumor. Therefore, nanoparticles reveal a great ability to target the solid tumor due to the enhanced permeability and retention (EPR) effect.

Maximizing the PS concentration in a solid tumor and controlling the ^1^O_2_ production within the cancer cells is a daunting challenge. NPs act as a promising PS carrier to the tumor through the passive EPR effect. Thus, a lower dose is required, which lessens side effects and provides a better therapeutic outcome compared to free PS treatment [88]. PEGylation of the surface of NPs with polyethylene glycol (PEG) to mask the NPs from the host immune system and increase their hydrodynamic size, which reduces renal clearance, is the most frequently used method to prolong NPs in blood circulation and increase drug accumulation at the tumor site [89]. Recently, surface modification by zwitterionic polymers has shown improved NP stabilization, longer retention in blood, and enhanced retention in the tumor compared to standard PEGylation, due to the super-hydrophilic nature and responsive properties of the pH [90,91,92]. Surface-modified nanocarriers with the EPR effect benefit PS delivery into tumors; however, only around 0.7% injected dose (ID) of nanocarriers are accumulated at the tumor site at the end [93]. Therefore, developing active targeting strategies has become an important direction to improve the efficiency of PS delivery.

#### 3.3.1. Antibodies on Nanocarriers for Specific Bioconjugation

As a general thought regarding active targeting, particular antibodies grafting onto the surface of NPs are able to facilitate the specific targeting to cancer cells and work toward increasing the nanodrug accumulation. Erlotinib, cyclic cRGDfK (cRGD) peptides, and aptamer-conjugated PSs showed specific targeting to epidermal growth factor receptor (EGFR)-positive cells, α_v_β_3_ integrin receptor-overexpressed cell lines, and protein tyrosine kinase 7 (PTK7) overexpressed tumor, respectively [94,95,96]. Tsai et al. (2018) used curcumin-encapsulated EGF-conjugated chitosan nanoparticles to recognize the EGFR-overexpressed MKN45 cells to kill in PDT [97]. In 2019, Zhang and coworkers developed photosensitizer protoporphyrin IX (PpIX)-conjugated peptide nanoparticles, which showed an excellent plasma membrane targeting capability [98]. Cho et al. (2020) developed a fucoidan-based nanocarrier that exhibits an excellent ability to conjugate with P-selectin-overexpressed neovascular endothelial cells around the tumor region, by which many PSs were transported into the tumor site for enhanced PDT [99].

#### 3.3.2. Mitochondria Targeting

Due to the short lifetime of ROS with a limited diffusion distance (<20 nm), and since mitochondria are critical intracellular organelles that generate energy to keep the cell alive and maintain growth, a promising strategy in PDT is to directly target mitochondria with nanocarriers and then destroy them with ROS. Triphenylphosphonium (TPP) is the most common mitochondrial-targeted agent. TPP has been modified onto the surface of numerous nanomaterials to achieve the aim of mitochondria-mediated apoptosis for cancer treatment [100,101]. Tetramethylrhodamine-5-isothiocyanate (TRITC) has been grafted onto upconversion nanoparticles (UCNPs) with graphene quantum dots (GQDs), which have the photosensitization feature, resulting in a good performance in mitochondria-targeted PDT [102]. The organic nanoparticles composed of chlorin derivatives developed by Liu et al. show an intrinsic ability of mitochondria targeting [103]. Yi et al. demonstrated that amphiphilic gemini iridium (III) complex-based nanovesicles are able to specifically target mitochondria for further PDT [104].

#### 3.3.3. Activation of Silent PSs

Aggregation-induced silencing of PSs could significantly reduce cytotoxicity during delivery, and their photoactivity could be switched on through an additional trigger, including H^+^, heat, enzymes, and remote interferences (microwave and ultrasound (US)). This approach not only shows enhanced efficacy in specific malignant cells but also restricts the area of ROS action to reduce the potential side effects. Li et al. showed that the aggregated zinc (II) phthalocyanine derivative entrapped on mesoporous silica nanoparticles (MSNs) could be dispersed through the trigger of albumin to enable photoactivity inside the cancer cell [105]. Typical thermal-responsive polymers were used to prepare activatable PS-loaded NPs, showing that the polymer’s physical expansion dramatically turns on the photoactivity of PSs at above 42 °C [106,107]. Yao et al. designed vesicular composite assemblies using block co-polymers with quinone linkage and PS moiety, which could be degraded by cytosolic NAD (P) H: quinone oxidoreductase isozyme 1 (NQO1) to exhibit the ability to specifically target NQO1-overexpressed cancer cells [108]. Several nanoplatforms have been developed, indicating that the silent state of PSs can be turned into an activated state under an acidic microenvironment [109,110,111]. The photoactivity of C_3_N_4_ quantum dots (QDs) producing vast amounts of ^1^O_2_ was triggered by microwave, showing an obvious benefit of limiting the area of efficacious treatment in a tumor in areas reached by the irradiation [112]. Zhang et al. showed a new strategy using US to collapse protein-based nanocarriers for further liberation of PSs and chemotherapeutic drugs for enhanced tumor-targeting PDT and chemotherapy [113].

#### 3.3.4. Cell Membrane-Camouflaged Nanocarriers

Surface engineering of nanomaterials by modifying aptamers, peptides, folic acid, and antibodies has the advantage of specifically targeting cancer cells to promote dose accumulation. Recently, as a progressive approach based on surface modification, coating cell membrane onto the nanomaterial to deliver PSs not only provides the active targeting function to cancer cells but also perfectly camouflages the PS as an innate biological substrate to reduce the recognition by macrophages, resulting in the maximum cumulative dose and minimum dose use and dark cytotoxicity [114,115,116]. Bidkar et al. (2020) developed transferrin (TF)-exposed cell membrane-coated PLGA NPs to co-carry DOX and PSs, showing excellent efficacy in TF receptor-overexpressed cancer cells in chemotherapy and PDT [116]. The cell membrane fragments from cancer cells were utilized to coat the PS-loaded nanocarriers that exhibited a distinct ability to target homologous cells [115].

#### 3.3.5. Magnetic Targeting

Co, Fe, Ni, iron oxide, and some ferrites are the most common materials to be widely used for the preparation of superparamagnetic nanoparticles. Recent reports demonstrated that using superparamagnetic NPs as PSs carriers provided the ability to specifically target the cancer cell through magnetic field attraction, and these NPs have potential use as contrast agents for magnetic resonance imaging (MRI) [117,118,119,120,121]. Talmoud et al. used γ-Fe_2_O_3_ NPs to load mTHPC, leading to a remarkably increased cumulative dose of PSs inside the tumor through the magnetic targeting [119]. Yan et al. (2018) directly applied PpIX to coat magnetic nanoclusters, revealing a high accumulation at the tumor, thus significantly enhancing the T_2_-weighted signals in MRI [120]. Wang et al. created magnetic nanobullets, composed of a body of disulfide-bridged m-SiO_2_ with Ce6 loading and a magnetic Fe_3_O_4_ head, which were used to conduct the combination therapy of PDT and alternating current magnetic-field-induced hyperthermia [121].

### 3.4. Additional Functions of Applying Nanocarriers in PDT

**Figure 4:**
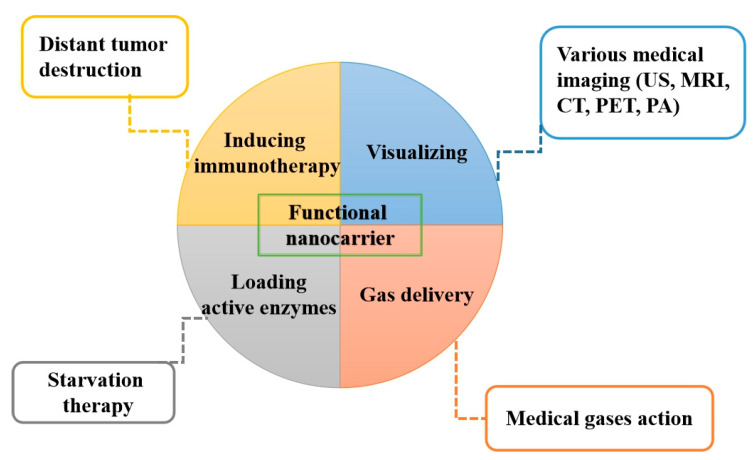
Nanoparticles can act as a tumor targeted carrier to deliver photosensitizers to a deep-seated tumor with additional functions. US: Ultrasound, MRI: Magnetic resonance imaging, CT: Computed tomography, PET: Positron emission tomography, PA: Photoacoustic.

With the advantages of functional NPs, the efficiency and efficacy of PDT have been further improved through additional actions from nanocarriers. Figure 4 summarizes additional functions from various NPs that improve the modern PDT applications in different ways.

#### 3.4.1. Imaging-Guided PDT Using Multifunctional Nanocarriers

Nanocarriers provide imaging functions as a means of monitoring PSs to ensure that they have been delivered into the cancer cells, ensuring not only excellent spatiotemporal selectivity to kill the cancer cells specifically but also minimal dark toxicity to normal cells during the drug transport. In recent years, numerous nanomaterials were developed to conduct imaging-guided PDT. Various nanocarriers containing the MnO_x_ component showed the capability of T_1_-weighted contrast imaging through Mn^2+^ release, which was obtained by MnO_x_ dissociation under the GHS or H_2_O_2_-mediated catalysis reaction in the TME [122,123]. Wang et al. (2020) designed fibronectin-targeting iron oxide nanocarriers to carry PSs, showing an excellent homing feature in triple-negative breast cancer (TNBC) treatment and an intrinsic ability in T_2_-weighted MRI [124]. Some particular nanoparticles with a high X-ray attenuation coefficient are suitable carriers to deliver PSs in computed tomography (CT) imaging-guided PDT [125]. The hyperthermia caused by photothermal nanomaterials upon light irradiation not only inflicts heat damage to the tumor but also produces sonic waves visualized in photoacoustic (PA) imaging. Two PA-guided PDT platforms using molybdenum oxide nanorings and iridium (III)-cyanine-based NPs have been established by the teams of Li and Yang [126,127]. Holmium (III)/ iridium(III) bimetallic complex NPs with a hollow structure exhibited distinct characteristics in the generation of singlet oxygen and typical US imaging owing to their cavity features [128]. PS-loaded nanocarriers with the properties of AIE or luminescence at the NIR range provided high-quality fluorescent imaging with the features of high spatial resolution and minimal interference by autofluorescence [129,130]. Xu et al. used mesoporous silica NPs to load PSs and incorporate with ^64^Cu-labeled peptides, and this technique may be feasible in positron emission tomography (PET) imaging-guided PDT [131]. Lee et al. showed that diethylenetriaminepentaacetic acid chelated Eu^3+^ and PSs-co-embedded liposomes with ^64^Cu-labeling, exhibiting an ultra-bright radioluminescence (RL) after ionizing radiation, by which good consequent imaging and PS activation was obtained [132]. Multimodal imaging-guided PDT has been achieved using some particular nanomaterials and combining various ideal imaging techniques such as US, PET, CT, MRI, PA, and luminescence, revealing perfect and precise tracking of PS-loaded nanocarriers [133,134,135].

#### 3.4.2. Enhancing Immunogenicity

In recent years, immunotherapy has attracted enormous attention due to its huge potential in cancer treatment, especially for its surprising efficacy in late-stage patients with distant or regrowing tumors [136,137]. A well-known medical strategy to conduct immunotherapy is injecting monoclonal antibodies to block the particular immunosuppressive pathways, thereby switching on the immune activity for tumor inhibition, a technique named checkpoint blockade immunotherapy (CBI). In current CBI, inhibitors are used to specifically block the expression of programmed cell death protein 1 (PD-1) on T-cells or its receptor PD-L1 on cancer cells [136]. However, immunotherapy showed limited efficacy for large primary tumors. Therefore, the combination strategy of a main PDT treatment for primary tumors and aiding immune response to kill the residual, metastatic, and recurrent cancer cells showed a potential improvement of pancreatic cancer treatment. Hu et al. used HA-modified Cu-porphyrin NPs to kill the primary tumor and subsequently to activate enhanced immunotherapy by using PD-L1 inhibitor [138]. Ce6-loaded immunoglobulin G (IgG, a type of immunologic adjuvant) nanocomposites with dual checkpoint blockade (PD-L1 and CTLA-4) have been used to treat solid tumors and prevent metastasis [139].

Several reports have shown the feasibility of PDT-induced immunotherapy. Chen et al. applied Ce6 and O_2_-encapsulated protein nanocarriers to treat the primary tumor in oxygen-supplied enhanced PDT under laser irradiation, resulting in the enhanced release of damage-associated antigens (TAAs) from dying cancer cells. These antigens, such as CRT, HMGB1, and ATP, induced the maturation of dendritic cells (DCs) and further elicited the action of T-lymphocytes and natural killer (NK) cells in lymph nodes as well as against distant tumors and lung metastasis [140]. The reports demonstrated that generating high amounts of ROS by PDT using PS-loaded nanocarriers not only led to immunogenic cell death (ICD) in the primary tumor to release damage-associated molecular patterns (DAMPs) but also induced proinflammatory M1-macrophage polarization to secret cytokine, which tended to cause an intense immune activation for distant tumor destruction [141,142]. Wang et al. developed new material N-doped carbon-silica nanocomposites (CSNs) with intrinsic ^1^O_2_-produced features (PS-free) that show good performance in photothermal and photodynamic therapies under light irradiation at 808 nm as well as provide immunoadjuvant properties to induce the TAAs and mature DCs [143]. Yu et al. tried to combine PDT and 3-MA as an autophagy inhibitor for the treatment of osteosarcoma, by which the autophagy was inhibited, triggering the upregulationof calreticulin (CRT) expression on the cancer cell membranes and reducing PD-L1 mono-ubiquitination, resulting in a synergistic immune response [144]. T-lymphocyte-associated protein 4 is a transmembrane receptor on T-lymphocytes with the functions of deactivation of T-lymphocytes and suppression of tumor immune effects. Xu et al. and Lin et al. injected anti-CTLA-4 after PDT, showing an effective inhibition of distant tumors [145,146].

#### 3.4.3. Regulating Metabolism of Cancer Cells by Starvation

In general, the cancer cells need to consume a large amount of glucose, thereby obtaining enough energy to sustain cell activity and proliferation. Some particular enzymes are able to trigger the essential substances’ degradation inside cancer cells to block the nutrient support through a method that has considerable potential in deep-seated tumor treatment called starvation therapy (ST) [147]. A highly important concern in ST is maintaining the enzyme’s natural activity after circulation in veins and internalization into cancer cells. Delivery of active enzymes to cancer cells by the nanocarrier is a useful and efficient approach to avoid the denaturation of these unstable proteins during the transport process as well as increase their accumulation in the tumor. Excellent results for the combination treatment of PDT and ST against tumors have been reported recently. Yu et al. applied Ce6 and glucose oxidase (GOx)-modified hollow MSNs encapsulating CPPO to treat lung cancer. In this system, GOx is able to decompose glucose into H_2_O_2_, and the CPPO is activated by this H_2_O_2_ to trigger the CRET-based PDT [148]. Zhu et al. used the GOx-loaded MSNs with PS-embedded lipid membrane shells to produce ROS and consume glucose inside cancer cells under irradiation at 730 nm, resulting in a synergistic ST and PDT treatment [149].

#### 3.4.4. Gas-Releasing Nanoparticles

**Figure 5:**
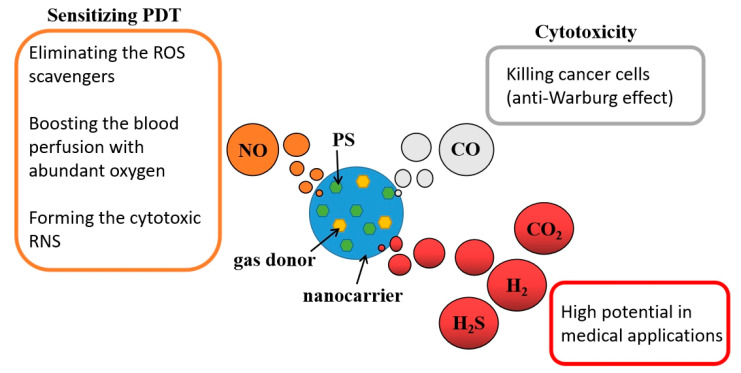
Various medical gases with unique functions could be delivered into the tumor site by nanocarriers to enhance PDT efficacy in tumor treatment.

Various medical gases such as nitric oxide (NO), hydrogen (H_2_), sulfur dioxide (SO_2_), hydrogen sulfide (H_2_S), carbon dioxide (CO_2_), and carbon monoxide (CO) are known to have benefits and bio-toxicity properties in practical bio-application [150]. Administration of medical gases in traditional gas therapy through gas exposure, directly inhalation, or injection approaches showed a high-risk of harm and serious side effects. Using nanotechnology, medical gas or gas donors can be carried to precisely target an area. The combination of gas therapy and PDT is a feasible strategy to augment the overall efficacy (Figure 5). Generally, the high amount of CO shows significant toxicity to cells through the anti-Warburg effect [151]. Ma et al. used fluorinated amphiphilic dendritic peptide (FADP) nanocarrier loading of Ce6 and CO releasing molecules (CORMs) to kill bacteria with ^1^O_2_ and CO [152]. Releasing NO as a cell signaling molecule in a deep-seated tumor could counteract the ROS scavenger activity of GSH, causing smooth muscle cell (SMC) relaxation to raise the blood perfusion with abundant oxygen supply and reacting with ROS to form cytotoxic reactive nitrogen species (RNS), thus resulting in multiple effects that improve the overall efficacy [151,153,154]. Wan et al. used a porous Zn-porphyrin nanocarrier to load L-arginine as a NO donor, presenting a feasible strategy of NO-sensitized PDT [155]. Yu et al. (2019) utilized PLGA vesicles to load hydrophobic TPP in polymer shells and hydrophilic NO donor sodium nitroprusside (SNP) in cavities. The SNP could be activated by glutathione and cysteine, which are rich in cancer cells, to generate NO that can inhibit mitochondria respiration, thus leading to a much lower oxygen consumption compared to no NO presence, consequently keeping more oxygen inside cells for ^1^O_2_ generation by PDT [156]. Other medical gases such as H_2_, CO_2_, and SO_2_ that are yet to be studied also play an important role in providing particular bio-functions, revealing high potential for use in enhanced PDT-related applications in the future (Figure 5).

## 4. Conclusions

In clinical trials, there is still a huge barrier to applying PDT to treat deep-seated tumors due to these unresolved limitations, including no light reaching deep-seated tumors, insufficient oxygen concentration in the TME, and insufficient PS accumulation in tumors. The approach using individual nanoparticles to carry PSs in PDT application has been developed and provides a potential opportunity to overcome these barriers in PDT tumor treatment. Some new strategies and techniques such as two-photon excitation, chemiluminescence (CL), lamp implantation, PLNPs, and use of X-rays for triggering showed an excellent ability to activate PSs at the deep-seated tumor site. Many feasible approaches, including oxygen delivery, endogenous H_2_O_2_ decomposition by enzymes or MnO_2_-induced catalysis, and intracellular water splitting, exhibited hypoxia relief in solid tumors and enhanced the efficacy of PDT. Surface-modified nanocarriers with the capability of active cancer cell targeting or mitochondria targeting revealed improved PS accumulation at specific malignant cells. Visualization of nanocarriers in medical images can help us to conduct imaging-guided PDT. Moreover, PDT combined with immunotherapy, starvation therapy, and gas therapy is able to activate immune response, weaken the cancer cells, and sensitize the ROS therapeutic effect, respectively. The next step and challenge of PDT is to validate the in vitro and in vivo efficacy in clinical studies. The important issues that need to be settled are the standardization the protocol of drug and light dosages in different platforms and the safety of nanoparticles. How to integrate different functional NPs into a single and simple system is another challenge. We believe a new platform that provides in situ actions, either individually or in combination with effective photoactivity of PSs, oxygen support, specific targeting and additional functions in imaging, enhancing immunogenicity, ST, and gas therapy will exhibit powerful performance in the treatment of deep-seated tumors in the future.

## Figures and Tables

**Table 1:** Summary of recent strategies using nanocarriers to resolve the limitations of practical photodynamic therapy (PDT) application.

Types of Nanomaterials	Particular Features	Functions	Reference
Au clusters	Two-photon absorption	Improving light penetration	[22,31,32]
ZnTPyP@TiO_2_ nanocomposites	Two-photon absorption	Improving light penetration	[33]
CaWO_4_ NPs, SrAl_2_O_4_:Eu^2+^ NPs and Cu NPs	X-ray-triggered persistent luminescence	Overcoming light penetration	[48,49,50]
ZnGa_2_O_4_:Cr	Persistent luminescence	Internal light in tumor site	[41,42,43,44]
Luciferase-exposed PLGA NPs	Bioluminescence	Internal light in tumor site	[40]
SPION	MR imaging and magnetic targeting	Imaging-guided PDT	[117,118,119,120,121]
Holmium(III)/iridium(III) bimetallic complex NPs	US imaging	Imaging-guided PDT	[128]
Zn-porphyrin-based nanoassemblies	NO release	NO-involved sensitized PDT	[155]
CORM-loaded FADP nanocarriers	CO release	Killing bacteria and ablation of biofilms	[152]
GOx-modified HMSNs	Decomposition of glucose	Starvation therapy	[148,149]
N-doped carbon-silica nanocomposites	Immunoadjuvant properties	Enhancing immunogenicity	[143]
TF-exposed RBC membrane-coated PLGA NPs	Targeting to TF receptor-overexpressed cancer cells	Enhancing PS concentration	[116]
Gemini iridium(III) complex-based nanovesicles	Mitochondria targeting	Enhancing PSs concentration	[104]
PpIX-conjugated peptide NPs	Plasma membrane targeting	Enhancing PSs concentration	[98]
Fe_3_O_4_@m-MnO_2_ NPs	Oxygen modulation and magnetic targeting	Hypoxia relief in TME	[83]
Carbon nitride (C_3_N_4_)	Water-splitting	Hypoxia relief in TME	[87]
V_2_O_5_ NPs	Peroxidase-like activity	Hypoxia relief in TME	[80]
pH-sensitive PFC-modified nanoparticles	Loading oxygen	Hypoxia relief in TME	[69]

## Data Availability

No new data were created or analyzed in this study. Data sharing is not applicable to this article.
